# Conformational Changes of α-Crystallin Proteins Induced by Heat Stress

**DOI:** 10.3390/ijms23169347

**Published:** 2022-08-19

**Authors:** Yu-Yung Chang, Meng-Hsuan Hsieh, Yen-Chieh Huang, Chun-Jung Chen, Ming-Tao Lee

**Affiliations:** 1 Life Science Group, Scientific Research Division, National Synchrotron Radiation Research Center, Hsinchu 30076, Taiwan; 2 Department of Biotechnology and Bioindustry Sciences, National Cheng Kung University, Tainan City 701, Taiwan; 3 Department of Physics, National Tsing Hua University, Hsinchu 30013, Taiwan; 4 Department of Biological Science and Technology, National Chiao Tung University, Hsinchu 300193, Taiwan; 5 Department of Physics, National Central University, Jhongli 32001, Taiwan

**Keywords:** α-crystallin, chaperone activity, circular dichroism, small-angle X-ray scattering

## Abstract

α-crystallin is a major structural protein in the eye lenses of vertebrates that is composed of two relative subunits, αA and αB crystallin, which function in maintaining lens transparency. As a member of the small heat-shock protein family (sHsp), α-crystallin exhibits chaperone-like activity to prevent the misfolding or aggregation of critical proteins in the lens, which is associated with cataract disease. In this study, high-purity αA and αB crystallin proteins were expressed from *E. coli* and purified by affinity and size-exclusion chromatography. The size-exclusion chromatography experiment showed that both αA and αB crystallins exhibited oligomeric complexes in solution. Here, we present the structural characteristics of α-crystallin proteins from low to high temperature by combining circular dichroism (CD) and small-angle X-ray scattering (SAXS). Not only the CD data, but also SAXS data show that α-crystallin proteins exhibit transition behavior on conformation with temperature increasing. Although their protein sequences are highly conserved, the analysis of their thermal stability showed different properties in αA and αB crystallin. In this study, taken together, the data discussed were provided to demonstrate more insights into the chaperone-like activity of α-crystallin proteins.

## 1. Introduction

Lenses are the primary units used to sense variations from environmental light. Lens proteins, crystallins, play major roles in maintaining the transparency and high refractive index in all vertebrate eye lenses [1,2]. Lens transparency is impaired by the formation of light-scatting particles obstructing the transmission of light; however, one of most common causes is aging [3,4]. The opacification or clouding of a lens is a major symptom of cataracts, which ultimately lead to a decrease in vision, or even blindness. Optical surgery for cataracts is the optimal way to rebuild the visual functions, but it is not affordable for low-income patients and is accompanied by surgery risks. Therefore, the development of non-surgical as well as preventive treatments for cataracts is inevitable for the increasing elderly population in the world [5]. Crystallins are the predominant soluble proteins in the lens, which are classified into distinct families: α-crystallin, β-crystallin, and γ-crystallin. α-crystallin is composed of two subunits, αA and αB, belonging to the small heat-shock protein family and that possess critical chaperone-like activity for the maintenance of protein homeostasis by recovering misfolding, aggregation, and suppression of thermally induced protein aggregation [6,7,8,9].

In the human lens epithelial cell line, α-crystallin proteins have the ability to inhibit cell death induced by heat stress [10]. In most mammalian lens, α-crystallins form a hetero-oligomeric complex of αA and αB crystallins with an association ratio of 3:1 [11]. The thermal behaviors, structural characteristics, and chaperone activity of α-crystallins have recently been widely studied over broad temperature ranges. α-crystallins were isolated from the lens, whereas the secondary and tertiary structure were changed and formed high-order molecular weights through heat treatment [12,13]. The conformational change was irreversible when the temperature was lowered [12]. Moreover, the chaperone activity showed no significant effect on structural changes in α-crystallins by heat stress [12]. After heating, small-angle X-ray scattering (SAXS) and dynamic light scattering (DLS) indicated that the conformation and shape of α-crystallins were variant, and the solvent-exposed surface was increased as it was responsible for enhancement of its chaperone activity [14]. Protein sequences of αA and αB crystallin share about 60% similarity for most mammalians [15,16]. Recent studies of recombinant αA and αB crystallin indicated that two α-crystallins form homo-oligomers in solution, but show different characteristics in thermostability. αA crystallin appeared clear and stable up to 100 °C in solution [17]. In contrast, when incubated at a high temperature, αB crystallin showed protein denaturing above 60 °C. The addition of αA crystallin to αB crystallin made the αB crystallin more stable at high temperatures [18].

However, small heat-shock proteins (sHsps) are abundant and ubiquitous in life. They are widespread in mammals, plants, and insects, as well as in microorganisms, and carry out diverse functions, such as cell development, protecting cells from apoptosis, stabilization of the cytoskeleton, and proteostasis [19,20]. As a member of the sHsp family, α-crystallins have been widely studied because of their high relevance to cataract formation in humans. Thus, α-crystallins are a good target for not only studying the mechanism of cataracts but also the chaperone activity of sHsp in different organisms.

In this study, circular dichroism (CD) was used to probe the change in secondary structure, and SAXS techniques were employed to determine the size of oligomers in human αA and αB crystallin with increasing temperature. Although the structural characteristics and thermostability of αA and αB crystallin were distinctive over 55 °C, two α-crystallins shared very similar behavior from 25 °C to 55 °C. Both of the α-crystallins demonstrated high ability in preventing aggregation of the substrate at 37 °C. However, although αA crystallin retained high chaperone activity at 55 °C, αB crystallin lost the ability to anti-aggregate. The resulting evidence of the structural characteristics and thermal properties of homo-oligomeric αA and αB crystallins and hetero-oligomeric α-crystallin holds major significance for their chaperone activity function in eye lens. Furthermore, we demonstrate a hybrid approach combining CD, SAXS, and aggregation assays for temperature effects on the structural characteristics and chaperone activity of α-crystallins, the important model sHsp.

## 2. Results

### 2.1. Circular Dichroism Shows Conformational Change Induced through Elevated Temperature

**Figure 1:**
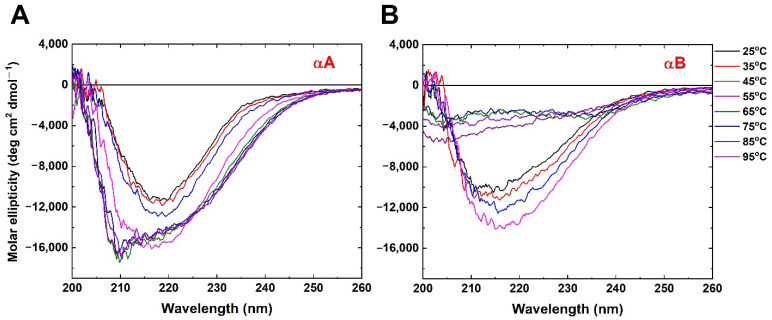
CD spectra of (**A**) αA and (**B**) αB crystallin from 2 °C to 95 °C in 20 mM phosphate buffer (pH 7.4, 2 mM NaCl).

To understand the biochemical and biophysical characteristics of αA and αB crystallins from humans, protein production was induced from *E. coli* as recombined proteins, His-tagged at the N-terminus. High purity of both αA and αB crystallins were purified from nickel-affinity and size-exclusion chromatography and analysis in this study. Far-UV circular dichroism (CD) was employed to monitor the secondary structure and characteristics of both α-crystallins at different temperatures (from 25 °C to 95 °C) (Figure 1). αA and αB crystallins showed comparable profile of far-UV CD spectra with characteristic minima detected at 217 nm and 212 nm at 25 °C, respectively (Figure 1). Upon stepwise rising temperatures from 25 °C to 55 °C, the signal of ellipticity became more pronounced in both α-crystallins, suggesting induced secondary structure content by heat [21] (Figure 1). Above 55 °C, the negative peak of the CD spectrum of αA crystallin retained its signal and shifted from 217 nm to 210 nm (Figure 1A). However, significant loss of ellipticity was observed from 55 °C to 95 °C in the αB crystallin (Figure 1B). Despite the conformation of αA crystallin existing more stably, αB was totally disrupted above 55 °C (Figure 1B). Taken together, both α-crystallins shared similar characteristics of structure below 55 °C, but differed significantly in thermal stability above 55 °C. More quantitative results extracted from CD spectra are shown in Table 1. The helical content of α-crystallins increases with temperature increasing, but the changes of secondary structure are within 10%. It is obvious that the difference in CD spectra between αA crystallin and αB crystallin was dominated by protein oligomerization rather than secondary structure change.

### 2.2. Thermal Denaturation Studies

**Figure 2:**
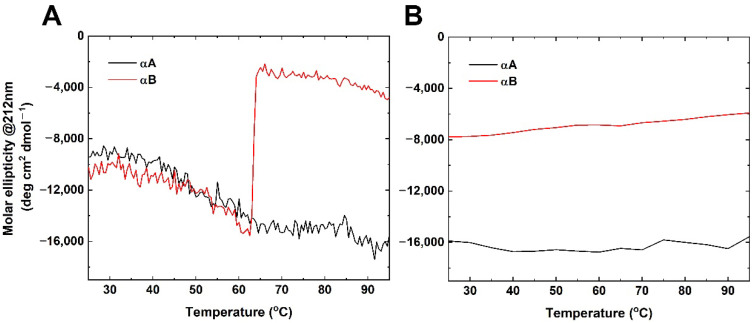
Representative (**A**) thermostability and (**B**) temperature reverse for αA and αB crystallin measured by circular dichroism. Circular dichroism signals at 212 nm were monitored at the rate of 1 °C/min from 25 to 95 °C and −5 °C/min from 95 to 25 °C, respectively.

Furthermore, CD was employed to investigate the thermostability of both α-crystallins. The CD signal at 212 nm was recorded when the temperature was continuously increased from 25 °C to 95 °C (Figure 2A). Interestingly, the solution of αA crystallin sample was clear but precipitation from the αB sample occurred in the cuvette at a high temperature. The conformational transition of αA crystallin was shown in a broad temperature range (35 °C to 65 °C) (Figure 2A). In contrast, the thermal denaturation studies showed that αB crystallin revealed a sharp and sigmoidal thermal denaturation profile with respect to temperature, reflecting the protein unfolding (Figure 2A). Moreover, both α-crystallins were shown to be irreversible when the sample was at 95 °C to 25 °C, under the same conditions (Figure 2B).

### 2.3. Enhancement of Chaperone-like Activity in αA but Loss of Function in αB Crystallin at High Temperatures

**Figure 3:**
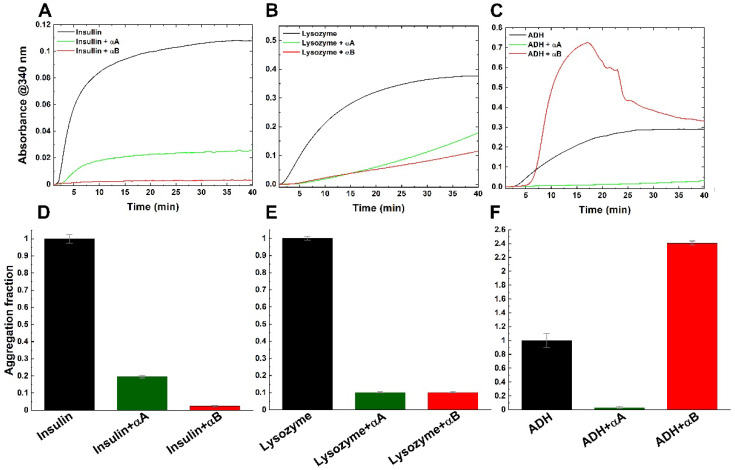
Chaperone-like activity of α-crystallins for the chemical- and heat-induced aggregation of target proteins. Assays were performed at 37 °C for insulin and lysozyme and 55 °C for alcohol dehydrogenase, ADH. Curves indicate the aggregation of insulin (**A**), lysozyme (**B**) or ADH (**C**) alone or with αA or αB crystallin. Chaperone activity was represented as aggregation fraction (**D**–**F**). Assays were performed in triplicate and the bars represent the mean ± SD.

It is well known that α-crystallin is a member of small heat-shock proteins functioning to bind unfolded substrates in response to stress and preventing their aggregation in the cell [22,23,24]. Conformational changes in a part of small heat-shock proteins have been studied in chaperone function and cell protection for heat and oxidative stress [25,26]. Although the properties of secondary structure and thermostability were disparate above 55 °C, both α-crystallins shared high-level similarity from 25 °C to 55 °C. To evaluate the chaperone-like activity of both α-crystallins at and above physiological temperature, different substrates were employed for aggregation analysis. To design the experiment, the ability of both α-crystallins in anti-aggregation was compared by measuring absorbance with the chemical-induced aggregation of two substrates at physiological temperature, insulin and lysozyme, and the thermal-induced substrate at 55 °C, alcohol dehydrogenase (ADH) (Figure 3A–C). For all experiments, samples were mixed at a molar ratio of 1:2 (α-crystallin/substrate) and heated to appropriate temperature, during which absorbance at 360 nm was recorded for 40 min. In the absence of α-crystallin, tree substrates significantly formed aggregation. For substrate insulin (37 °C), αA crystallin effectively exhibited an ~80% decrease in aggregation, and αB crystallin showed greater chaperone-like activity with a ~95% reduction in aggregation (Figure 3D). Moreover, both α-crystallins also provided high efficiency of ~90% against the aggregation of lysozyme (37 °C) (Figure 3E). Similarly, αA crystallin revealed a strong anti-aggregation ability with αB crystallin at physiological temperature. Heating it to 55 °C, αA crystallin totally inhibited the thermal-induced aggregation of ADH (Figure 3F). However, αB crystallin almost lost the chaperone-like activity at the high temperature (Figure 3F).

### 2.4. Heat-Induced Changes in Conformation and Size by SAXS Analysis

**Figure 4:**
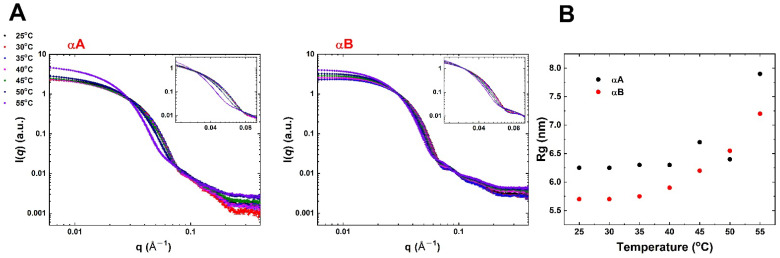
(**A**) SAXS profile of α-crystallins at various temperatures. Upon increasing temperature, the scattering pattern significantly changed in *q* range between 0.03 and 0.1 Å^−1^ (inset), indicating that morphologies changed with increasing temperature. (**B**) Evolution of the radius of gyration (*R*_g_) of α-crystallins as a function of various temperatures. The effects of temperature rising on the structure of α-crystallins are reflected in the increase in *R*_g_.

The SAXS technique is a useful experiment for providing the overall size, conformation, and shape of biological macromolecules in solution [27,28,29,30]. Since efforts for the crystallization of α-crystallins have been unsuccessful, we collected synchrotron SAXS data for obtaining information of overall structure and radius of gyration (*R*g) at different temperatures (25 °C to 55 °C) (Figure 4). High purity and homogeneity of protein samples (250 μM) for SAXS experiments were prepared using an SEC-FPLC system and used for SAXS data collection at synchrotron beamline (TLS BL23A1, Hsinchu, Taiwan). Scattering curves of α-crystallins in solution revealed that the shape changed at 45 °C for αA and temperature increased for αB, especially at *q* from 0.03 to 0.1 Å^−1^ (Figure 4A). The *Rg* was extracted from scattering curves using the PRIMUS program. Meanwhile, the analyzed *Rg* of αA was 6.3 nm to 8.0 nm, and αB was 5.7 nm to 7.2 nm from 25 °C to 55 °C (Figure 4B). SAXS results suggested that the conformational change, size enlargement, and altered shape of both α-crystallins were induced by increasing temperature.

## 3. Discussion

α-crystallin, a member of the small heat-shock protein (sHsp) family, is expressed in high concentrations in ubiquitous mammalian lenses and is known to exhibit chaperone activity for the aggregation resistance of other proteins in response to harsh stresses including pH, temperature, and ion strength [22]. Two isoforms of α-crystallin have been identified to date—αA and αB crystallins with 57% sequence identity, which are only expressed in lens and non-specific tissues, respectively [18,23]. Expressed together in the lens, the native forms of αA and αB crystallin preferentially formed a 600–800 kDa hetero-oligomeric complex in a 3:1 ratio [23] and exhibited more thermal stability through intermolecular exchange between αA and αB crystallin [17]. The topic of the relationship between structural variation through heat induction and chaperone-like function has numerous reports for the understanding of heat-stress response [31,32,33,34,35,36,37]. Hetero-oligomeric native α-crystallin showed that the secondary structure was changed, and overall conformation was preserved from 25 °C to 95 °C [12,38]. In this study, biophysical and biochemical properties of the homo-oligomeric form in αA and αB crystallins showed that these properties were distinct from the temperatures of 55 °C to 95 °C, but similar between 25 °C and 55 °C (Figure 1). The characteristic of thermostability in homo-oligomeric αA crystallin shared unanimous behaviors with hetero-oligomeric native α-crystallin rather than αB crystallin.

A mild heating model for age-related cataract studies showed that the membrane environment of human lens epithelial cells (LECs) appears more fluid. Zhang et al. indicated that α-crystallins were accumulated around the nucleus at LECs after mild heating to 50 °C [39]. Heat treatment contributed to αA-crystallin-facilitated insertion into the membrane [40]. Additionally, the secondary and tertiary structures of α-crystallins were mainly changed below 60 °C [37]. Another possible mechanism for the accumulation of α-crystallins around the nucleus and interaction with membrane is that the conformation of α-crystallins was altered through heating of the proteins.

Both αA and αB crystallins maintained secondary structure and stability below 60 °C (Figure 1). Das et al. reported that native α-crystallin not only formed a high-molecular-weight (HMW) aggregation structure, but also increased chaperone activity at 60 °C [37]. Even though αB crystallin almost lost the anti-aggregation function, αA crystallin still reserved high chaperone activity at 55 °C (Figure 3F). The chaperone activity of α-crystallins at high temperatures was mainly contributed by αA crystallin.

In this study, SAXS data indicated that the shape and size of both αA and αB crystallins were changed in response to heat stress (Figure 4). Previous research showed the structure of α-crystallin in a central cavity under 42 °C [14]. Furthermore, the central cavity of α-crystallin disappeared with structure rearrangement as well as surface area increment above 42 °C [14]. Importantly, chaperone activity of αA crystallin increased with the heat-induced structural changes rather than αB crystallin. Overall, the structural characteristics and thermal property of homo-oligomeric αA and αB crystallins and hetero-oligomeric α-crystallin were of major significance for the function of chaperone activity. Recent EM and X-ray research has evidenced that hetero-oligomeric α-crystallin, as well as the enhancement of the function of α-crystallin during its dissociation, is significant for the function of α-crystallins [41]. This is also consistent with our conclusion mentioned above.

In conclusion, the effects of temperature on the structure of α-crystallin were highly correlated to its function of chaperone activity. Furthermore, αA crystallin has higher thermal stability than αB crystallin. It is well-known that X-ray crystallography and cryo-EM are powerful techniques to determine the structure of protein and build the correlation between structure and function. Unfortunately, neither of them are feasible for temperature-dependent measurements. Furthermore, a recent structural study that used EM and X-ray concluded that structural–functional studies of α-crystallins have been carried out under far-from-native conditions, and they cannot adequately reflect the features of the functioning of α-crystallin in vivo [41]. In this study, we carried out SAXS and CD measurements of αA crystallin and αB crystallin in solution with increasing temperature and correlated structural evidence to their function of chaperone activity. The rare structural information of temperature dependence for α-crystallin is helpful to understand the mechanism of cataract formation.

## 4. Materials and Methods

### 4.1. Cloning, Protein Expression, and Purification

The open reading frames of full-length αA and αB genes were amplified by PCR, containing NdeI/XhoI restriction sites, and cloned into the pET28 vector with the 6xHis tag at the N-terminus. For protein production, the expression constructions were transformed into *E. coli* BL21(DE3) competent cells, and the cells were cultured overnight with LB broth medium containing 50 μg ml^−1^ kanamycin at 37 °C. The cells were grown to an OD_600_ and reached 0.6, and protein expression was induced by adding a final concentration of 0.8 mM IPTG at 37 °C for 4hr. After induction, the cells were harvested at 6800× *g* for 15 min, and the pellets were resuspended with buffer A (20 mM phosphate buffer, pH 7.4 and 150 Mm NaCl). The cells were disrupted by sonication on ice and centrifuged at 14,000× *g* for 20 min at 4 °C. 

For purification, the cleared supernatant was applied onto Ni-NTA agarose (Qiagen). Nonspecific binging proteins were washed in buffer B (20 mM phosphate buffer, pH 7.4, 150 Mm NaCl and 150 mM imidazole), and the target-bound proteins were eluted in buffer C (20 mM phosphate buffer, pH 7.4, 150 Mm NaCl and 500 mM imidazole). To obtain higher purity, the fraction-containing target proteins were applied to a HiPrep Sepharcyl S-300 HR 16/60 size exclusion column and eluted with buffer D (20 mM phosphate buffer, pH 7.4, 150 mM NaCl). The fraction of peak was analyzed by SDS-PAGE and stained with Coomassie Blue.

### 4.2. Circular Dichroism Spectroscopy

CD spectra were monitored in a JASCO J815 spectrometer equipped with a Peltier temperature controller (PTC-423S) in a 1mm path length cuvette. Far-UV CD experiments were performed using a protein concentration of 20 μM (20 mM phosphate buffer, pH 7.4 and 2 mM NaCl) in the range of 200 nm to 260 nm by three wavelength scans at a rate of 20 nm/min. To assess the thermal stability, the signal of ellipticities at 212 nm was monitored from 25 °C to 95 °C at a rate of 1.0 °C/min. All the CD measurements for the thermal denaturation were repeated three times. The results are reproducible. The estimated contains of secondary structure were calculated using the software in DichroWeb [42] for the dataset [43].

### 4.3. Chaperone Activity Assay

Lysozyme, insulin and alcohol dehydrogenase (ADH) were purchased from Sigma. 

Protein lysozyme and insulin (concentration of 100 μM) and alcohol dehydrogenase (300 μg) were dissolved in buffer A as substrates and mixed with αA and αB (at concentrations of 50 μM) at 37 °C and 55 °C under different conditions, respectively. The lysozyme and insulin were incubated with 20 mM DTT for aggregation. The aggregation assay was monitored for 40 min at 360 nm.

### 4.4. Small-Angle X-ray Scattering (SAXS) Measurements

Both αA and αB crystallins were purified from the size-exclusion chromatography and prepared at a 0.25 mM concentration in buffer A for SAXS experiments. Measurements of SAXS were performed at the BL23A1 SWAXS endstation of Taiwan Light Source, National Synchrotron Radiation Research Center (NSRRC), Taiwan, with an X-ray beam of 15.0 keV (wavelength λ = 0.8267 Å) and at a sample-to-detector distance 3110 mm. The scattering intensities were recorded by the Pilatus-1MF detector of area 169×179 mm^2^ and pixel resolution 172 μm, covering the momentum transfer Q up to 0.4 Å^−1^, which was calibrated with a standard sample of silver behenate. Q=4πsinθ/λ, where 2θ is the angle of scattering. The background of the solutions without protein measured under identical conditions as for the sample was subtracted from the *I(Q)*. The radius of gyration (*R*g) was obtained from equating the initial slope of ln[I(Q)] to $-Q^{2}{R_{g}}^{2}/3\;$(Guinier plot) using PRIMUS [44].

## Figures and Tables

**Table 1:** Secondary structure contents of α-crystallins as a function of temperature extracted from CD spectra in Figure 1.

	αA Crystallin			αB Crystallin		
	α-Helix *	β-Sheet **	Unordered	α-Helix *	β-Sheet **	Unordered
25 °C	0.54	0.24	0.22	0.53	0.29	0.18
35 °C	0.51	0.27	0.22	0.52	0.32	0.16
45 °C	0.48	0.31	0.21	0.54	0.28	0.18
55 °C	0.50	0.30	0.20	0.51	0.30	0.19
65 °C	0.51	0.32	0.17	0.61	0.18	0.21
75 °C	0.52	0.30	0.18	0.61	0.21	0.18
85 °C	0.55	0.27	0.18	0.61	0.24	0.15
95 °C	0.54	0.27	0.19	0.64	0.22	0.14

* α-Helix = Helix1 + Helix2. ** β-Sheet = Strand1 + Strand2 + Turns.

## Data Availability

Not applicable.
